# Lectures on Renal & Urinary Diseases

**Published:** 1897-09

**Authors:** 


**Lectures on Renal & Urinary Diseases.** By Robert Saundby, M.D. Second Edition. Pp. xii., 434. Bristol: John Wright & Co. 1896.

These lectures, first issued as two separate works, are now revised and published in a single volume with additions, including a special section on stone in the kidney, hydronephrosis, pyelitis, and hæmoglobinuria.

Dr. Saundby's views are so clear, and his theories so well expressed, that we heartily welcome the reappearance of the lectures in this handy form. In spite of the brief, terse style, the number of small but important details given on every page show that the author is a master of his subject and of the art of description. Still, on some subjects we should have liked a fuller expression of his views. This is the case particularly in the new paragraphs on calculus and hæmoglobinuria, where he hardly does himself and his readers justice. The importance of the diagnosis of kidney affections, other than Bright's disease, is so great that every help such as the situation and character of pain and of the tenderness, the microscopic and other aids, must be considered. Thus the diagnosis of tuberculosis is hardly mentioned, though he elsewhere refers to the pseudo-bacillus which is so misleading in some instances. Recent investigations seem to prove that this is the smegma bacillus, and curiously enough it gives all the reactions of Koch's bacillus to ordinary stains, but can be decolourised by ten minutes immersion in alcohol. In this way alone can distinction be made between what is a harmless parasite and the chief agent in tubercular disease. The progress of modern surgery has increased the importance of an early recognition of this affection, which is often confined to the kidney alone. Indeed the surgeon is now offering his aid in the treatment of acute and subacute Bright's disease, where Mr. Reginald Harrison claims that an incision for the relief of inflammatory tension may sometimes put an end to suppression of urine and prevent the degenerative changes which too often follow the acute stage.

The chapter on urinary testing is a very valuable one, the more so that it is not too prolix, though in the tests that are given the greatest care has been taken to call attention to many neglected precautions. The author finds that the ferrocyanide test is vitiated by its throwing down albumose,[1] and denies, we think wrongly, the greater delicacy of the picric acid test for albumen, which has been regarded as one of its chief merits. Though errors have often been made from the cloud of mucin it precipitates, many observers believe that a smaller amount of albumen can be detected by it than by nitric acid. The bare statement that the excretion of uric acid is diminished by salicylate of soda is true, but misleading, for it is well known that Haig and others use it to obtain a greatly increased output in the form of a soluble salicylurate. This amounts to more than twelve times the normal according to Haig, while Bohland showed that this excretion is accompanied by a remarkable leucocytosis, indicating, as he thinks, an increased formation. A fuller account of Ehrlich's diazo-reaction would seem desirable if mentioned at all.

Piperazine has been tried and found wanting by Dr. Saundby, but we find no reference to the formalin compound, urotropine, which appears to be a powerful antiseptic for the urinary tract, as well as a diuretic and uric acid solvent. Putrid or ammoniacal urine is, it is said, rapidly rendered healthy by it.

By "dilution of the blood," cursorily mentioned in the treatment of uræmic coma, may be meant either simple injection of saline fluid, or the combined injection and bleeding which has been so much advocated of late.

We are glad to see that the view is taken that uræmia is a group of affections produced by different toxic agents, many of which are absorbed from the intestines, while evidence is given against the theory of cerebral œdema. We may add that Leonard Hill's investigations on the cerebral circulation seem also to negative that of spasm of the vessels, for he finds no proof of any vaso-motor mechanism in the brain at all. Thus, it has been remarked, we are brought to the view of a direct action of the toxins on the cortex as causing the convulsions and other phenomena.

The discussion of cardio-vascular changes is even more interesting. The conclusions are that cardiac hypertrophy is met with in all forms of Bright's disease, including acute nephritis, but is most common in the contracting kidney. It is due to (*a*) stimulation of the heart to increased activity by the presence of non-eliminated poisonous matter in the blood, and (*b*) increased capillary resistance. The latter may be ascribed to alterations in the density of the blood plasma. The high tension pulse is due to the greater energy of the heart and greater capillary resistance combined.

The importance of tube-casts for diagnosis and prognosis is still maintained by Dr. Saundby, in spite of their occasional appearance in health and in other diseases, and with respect to

1 Elliott maintains that this can be avoided if a little acetic acid be only added after the urine is mixed with the ferrocyanide.

the formation of hyaline casts his present conclusion is that they may arise either by metamorphosis of epithelial ones, or by coagulation of transuded plasma, or by secretion from the epithelial cells. The broader ones which are derived from epithelial forms by metamorphosis are, like them, produced only where the inflammation is of a severe type. We do not discover any reference to the use of the centrifuge, which renders our examination for casts much more exact and certain.

There is much to be said for and against the classification of the various forms of Bright's disease which has been adopted, but probably our knowledge is not yet ripe for any perfect arrangement. The three types of infective, lithæmic, and obstructive nephritis represent three groups fairly distinct in character, though almost any clinical or anatomical symptom may be found indifferently in cases of either type. The cases included in the first two classes are produced by toxins, both various and partly unknown, and the distinction would seem to be that the lithæmic type is one where the disease attacks, first, the cardio-vascular system, causing increased diuresis, and later on the kidneys; while in the infective class the kidney is first affected by the poison. Still, it may be doubted whether there is a primarily diminished diuresis in all these cases except the lithæmic. The extremely brief chapter on obstructive disease may give the reader an impression that puerperal nephritis is due entirely to pressure, in spite of the highly toxic character of the urine and other evidence to the contrary.

The lectures devoted to Diabetes are the most delightful part of the book. The history, distribution, and causes of the disease are admirably arranged and ably discussed, so that we only come to the end with a wish for more. The recent work of Pavy and Minkowski is noticed; but in fact the true pathology is so uncertain, in spite of the great amount of recent work, that perhaps the most valuable section is that on gaps in our knowledge. On the whole Dr. Saundby takes the view that the balance of evidence is in favour of the theory that over-production by the liver is the cause of diabetes, and that this is an excess of its normal vital function. He has quite given up the use of gluten bread, as it contains so much starch, and is both dear and unpalatable. In place of it, when a strict diet is necessary, he gives Callard's "brown loaf" and biscuit food, or others made with cocoa-nut or almond flour, to which a little gluten or aleuronat is added. The use of the cheap cocoa-nut meal deserves to be widely extended, though there is a slight difficulty in cooking it.

The microscopic drawings are perhaps hardly up to the average, but the printing and appearance of the book generally leave nothing to be desired, and every medical practitioner will find it a valuable work of reference.

